# Support vector machine based aphasia classification of transcranial magnetic stimulation language mapping in brain tumor patients

**DOI:** 10.1016/j.nicl.2020.102536

**Published:** 2020-12-24

**Authors:** Ziqian Wang, Felix Dreyer, Friedemann Pulvermüller, Effrosyni Ntemou, Peter Vajkoczy, Lucius S. Fekonja, Thomas Picht

**Affiliations:** a Department of Neurosurgery, Charité – Universitätsmedizin Berlin, Berlin, Germany; b Cluster of Excellence: “Matters of Activity. Image Space Material”, Humboldt Universität zu Berlin, Berlin, Germany; c Freie Universität Berlin, Brain Language Laboratory, Department of Philosophy and Humanities, Berlin, Germany; d University of Groningen, Department of Neurolinguistics, Groningen, The Netherlands

**Keywords:** AAL, automated anatomical labeling, AAT, Aachen aphasia test, AUC, area under receiver operating characteristic curve, BAS, Berlin aphasia score, ER, error rate, IQR, interquartile range, Machine Learning, ML, MNI, Montreal neurological institute, RFE, recursive feature elimination, ROC, receiver operating characteristic, ROI, region of interest, rTMS, repetitive transcranial magnetic stimulation, SVM, support vector machine, TMS, transcranial magnetic stimulation, VAS, visual analogue scale, Transcranial magnetic stimulation, Language, Support vector machine, Machine learning, Glioma

## Abstract

Repetitive TMS (rTMS) allows for non-invasive and transient disruption of local neuronal functioning. We used machine learning approaches to assess whether brain tumor patients can be accurately classified into aphasic and non-aphasic groups using their rTMS language mapping results as input features. Given that each tumor affects the subject-specific language networks differently, resulting in heterogenous rTMS functional mappings, we propose the use of machine learning strategies to classify potential patterns of rTMS language mapping results. We retrospectively included 90 patients with left perisylvian world health organization (WHO) grade II-IV gliomas that underwent presurgical navigated rTMS language mapping. Within our cohort, 29 of 90 (32.2%) patients suffered from at least mild aphasia as shown in the Aachen Aphasia Test based Berlin Aphasia Score (BAS). After spatial normalization to MNI 152 of all rTMS spots, we calculated the error rate (ER) in each stimulated cortical area (28 regions of interest, ROI) by automated anatomical labeling parcellation (AAL3) and IIT. We used a support vector machine (SVM) to classify significant areas in relation to aphasia. After feeding the ROIs into the SVM model, it revealed that in addition to age (w = 2.98), the ERs of the left supramarginal gyrus (w = 3.64), left inferior parietal gyrus (w = 2.28) and right pars triangularis (w = 1.34) contributed more than other features to the model. The model’s sensitivity was 86.2%, the specificity was 82.0%, the overall accuracy was 85.5% and the AUC was 89.3%. Our results demonstrate an increased vulnerability of right inferior pars triangularis to rTMS in aphasic patients due to left perisylvian gliomas. This finding points towards a functional relevant involvement of the right pars triangularis in response to aphasia. The tumor location feature, specified by calculating overlaps with white and grey matter atlases, did not affect the SVM model. The left supramarginal gyrus as a feature improved our SVM model the most. Additionally, our results could point towards a decreasing potential for neuroplasticity with age.

## Highlights

• First study using machine learning to classify rTMS language mapping results in tumor patients.
• Analysis of 90 patients reveals rTMS-induced error patterns in aphasic/non-aphasic patients.
• Increased vulnerability of right pars triangularis to rTMS in aphasic patients with left gliomas.

## Graphical abstract

**Figure:**
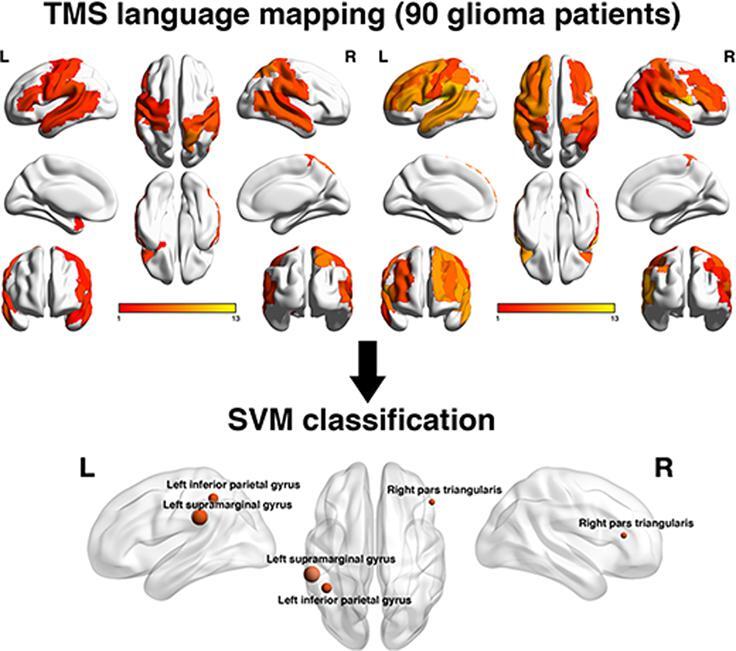

## 1 Introduction

10 years ago, rTMS was introduced by our group as a tool for planning brain tumor surgery based on individualized cortical language mapping (Lioumis et al., 2012, Picht et al., 2013). Nevertheless, its clinical reliability and usefulness for cognitive mapping remains debatable to this day (Bahrend et al., 2020, Schwarzer et al., 2018). We have therefore retrospectively analyzed our rTMS language mapping results of the last decade to classify aphasic and non-aphasic glioma patients using machine learning.

Regarding language, it has been postulated that different levels of language processing engage various brain areas, with different left-hemispheric streams being responsible for production and comprehension (Hickok and Poeppel, 2007, Price, 2012). Additionally, it has been suggested that processes such as syntax and semantics are supported by networks with different levels of laterality (Friederici, 2011). Moreover, current models on the neural bases of phonological and semantic representation propose neuronal assemblies distributed over bilateral but differentially lateralized perisylvian as well as extrasylvian sensorimotor and multimodal areas (Pulvermuller, 2018).

In addition to the organization of language in large-scale distributed networks (Duffau, 2014, Mesulam, 1990, Pulvermuller and Fadiga, 2010), language mapping is also challenged by the observation that individual language networks appear to be volatile in the sense that in the presence of an expanding brain tumor their dynamic processing patterns are subject to plasticity, both acute and long-term (Duffau, 2015, Duffau et al., 2003). Plasticity enables functional redistribution within remote networks and plays a central part in recovery after brain injury (Duffau, 2005, Duffau, 2015, van Dokkum et al., 2019).

To analyze the complex language network in relation to aphasia and rTMS object naming results, a promising possibility is the use of machine learning (ML). ML has better predictive power than traditional statistical methods. Compared to traditional statistics, ML focuses on finding patterns in rich and unwieldy data, and can also be used to infer data (Bzdok et al., 2018). Among ML methods, linear support vector machines (SVM) can be easy-to-interpret since they can learn linear discriminant functions and assign weights to features in the input space (Rosenbaum et al., 2011). Due to abovementioned advantages, SVMs have been widely used for various disease-related data (Cuingnet et al., 2011, Deo, 2015, Kannel et al., 1975). In addition, SVM has proven to outperform classical correlation methods, for example in gene selection biologically relevant to cancer (Guyon et al., 2002). SVM maximizes the classification margin and is more robust over more traditional approaches of analyses, for example multiple logistic regression, especially when a large feature space is considered for analysis and data is unbalanced (Christodoulou et al., 2019). This study is the first to analyze TMS data of tumor patients with an SVM classifier.

Our aim was to use machine learning to retrospectively investigate rTMS language mappings and classify aphasic and non-aphasic patients in rich feature space, consisting of individual regions’ specific rTMS language mapping error rates as well as patients’ lesion profiles and clinical data.

## 2 Methods

### 2.1 Patient cohort

We identified 296 patients in our prospectively collected database who received preoperative rTMS language mappings for left perisylvian brain tumors since 2010. Of these, 218 underwent the standardized consensus protocol (Krieg et al., 2017). After stratifying for glioma only (n = 147) and completeness of data including formal language testing (AAT/BAS), 90 patients were included into the study (41 female, 49 male, mean age 48.86 ± 14.12, age range 21–82, 12 WHO II°, 42 WHO III°, 36 WHO IV°). Patient characteristics are shown in Table 1. Handedness was determined using the Edinburgh handedness inventory (Oldfield, 1971). The exclusion criteria were: 1. Frequent generalized seizures (more than one per week); 2. Aphasia with more than 28% error rate in the baseline object naming task, identified as a reliability threshold in a previous study (Schwarzer et al., 2018); 3. Multicentric gliomas, and 4. Left-handedness.

**Table 1:** Demographics and neuropathological overview of the patient cohort.

	Non-aphasic patients	Aphasic patients	*p*
**Demographics**			
Trial size	61 (68%)	29 (32%)	
Age*	45.13 ± 12.84	56.69 ± 13.64	0.000018
Female	31 (51%)	10 (34%)	0.272
Male	30 (49%)	19 (66%)	0.272
Tumor volume** (cm^3^)	46.95 ± 45.31	47.17 ± 41.31	0.986
Right handedness	61(100%)	29(100%)	1
Time since tumor diagnosis (days)	2.23 ± 19.65	2.24 ± 8.33	0.988
**Tumor location**			
Frontal	25 (41%)	9 (31%)	0.498
temporal	21 (34%)	18 (62%)	0.025
Parietal	9 (15%)	1 (3%)	0.158
Insular	6 (10%)	1 (3%)	0.422
**Glioma WHO grade**			
Glioma II	11 (18%)	1 (3%)	0.0944
Glioma III	31 (51%)	11 (38%)	0.358
Glioma IV	19 (31%)	17 (59%)	0.024
**Error rate**			
Overall ER (%)	4.37 ± 2.93	8.43 ± 4.92	0.000004
Left ER (%)	4.55 ± 3.06	8.87 ± 4.66	0.000001
Right ER (%)	5.01 ± 3.67	6.71 ± 4.93	0.232

Values shown are M ± SD or n (percentage).

ER: Error rate (amount of positive TMS stimulations divided by the sum of positive and negative TMS stimulations).

*Age at the time of diagnosis.

**Tumor size was measured within 7 days before the TMS examination and BAS.

### 2.2 Ethical standard

The study proposal is in accordance with ethical standards of the Declaration of Helsinki and was approved by the Ethics Committee of Charité - Universitätsmedizin Berlin (#EA1/016/19). All patients provided written informed consent for all medical evaluations and treatments within the scope of the present study.

### 2.3 Data acquisition

#### 2.3.1 In-house data

MRI data were acquired using a Siemens 3T Skyra system (Erlangen, Germany) at Charité – Universitätsmedizin Berlin, Department of Neuroradiology. T1 weighted images were acquired with TR/TE/TI 2300/2.32/900 ms flip angle = 8°, field of view (FOV) = 230 × 230 mm^2^, matrix size 256 × 256, 192 sagittal slices, 1 mm isotropic resolution.

#### 2.3.2 Aphasia grading

The severity of aphasia was assessed preoperatively using the Berlin Aphasia Score (BAS). The BAS is used and developed by physicians of the Charité – Universitätsmedizin Berlin and adapted from the Aachen Aphasia Test (Huber et al., 1984). The test classifies patients into 4 categories (Picht et al., 2013, Schwarzer et al., 2018): 0 = no aphasia (61 patients), 1 = mild aphasia (18 patients), 2 = moderate aphasia (8 patients), 3 = severe aphasia (3 patients). All patients with a BAS score of 0 were grouped into the non-aphasic cohort, others were classified as aphasic patients. A Spearman's rank-order correlation was performed to determine the correspondence between BAS and AAT T-scores for a subset of patients under investigation (n = 60) for which both measurements were available. AAT subtests included in this version were Token Test, Verbal Repetition, Naming and Language Comprehension. BAS results were correlated to a composite score across all subtests.

#### 2.3.3 rTMS language mapping

Navigated rTMS language mapping was performed with nTMS eXimia NBS version 3.2.2, Nexstim NBS 4.3 and NexSpeech module (Nexstim Oy, Helsinki, Finland). The baseline naming performance of 150 black-and-white drawings of everyday objects was assessed prior to rTMS (M = 85.5, SD = 28.6, Min = 35, Max = 149). The images applied were provided by the Nexstim NexSpeech software. During baseline testing, the dataset was presented to the patients twice in the absence of any stimulation, while being video-recorded. In case errors were still made in the second baseline, the dataset was presented a third time. Only images remaining after the final baseline testing with correct responses were used for rTMS mapping, which covered the perisylvian cortex of both hemispheres (Schwarzer et al., 2018). The resulting set of pictures after baseline correction was presented in a randomized order.

The required individual stimulation intensity was determined prior to stimulation of language-relevant areas (Groppa et al., 2012). Each individual's responsiveness to TMS stimulation was determined by measuring the resting motor threshold (RMT) using the 5/10 method over the primary motor cortex of the respective hemisphere for the first dorsal interosseus muscle of the contralateral hand (Rossini et al., 2015). Consequently, language mapping was performed with 1-s trains of rTMS at 100% of the RMT. If the calculated cortical electric field was less than 50 V/m, the stimulation intensity was increased accordingly (Krieg et al., 2017). In case no stimulation effects on naming were observed by the examiner during the initial 20–30 rTMS trains, the parameters were modified to increase difficulty: first, shorter inter-image intervals (4–2.5 s), and, if still ineffective, the picture display time was decreased (1000–700 ms). Further, if still ineffective, different frequencies (7 Hz, 10 Hz) were tested (Hauck et al., 2015, Schwarzer et al., 2018). The objects remaining from the baseline were presented to the patient in randomized order. Each patient underwent one rTMS mapping session during which cortical areas were targeted based on the location and size of the tumor as well as the aphasia status and performance of the patient. For each hemisphere, 150–250 stimulations over 50–80 distributed sites were administered. Each spot was stimulated at least 3 times. If a positive effect was found during the examination, the respective spot was stimulated repeatedly to assess the reproducibility of the effect (Schwarzer et al., 2018). The degree of discomfort or pain during the mapping was evaluated with the visual analogue scale (VAS). The rTMS coordinates were exported as text files for subsequent analysis and spatial normalization.

#### 2.3.4 Spatial normalization & anatomical labelling

**Fig. 1:**
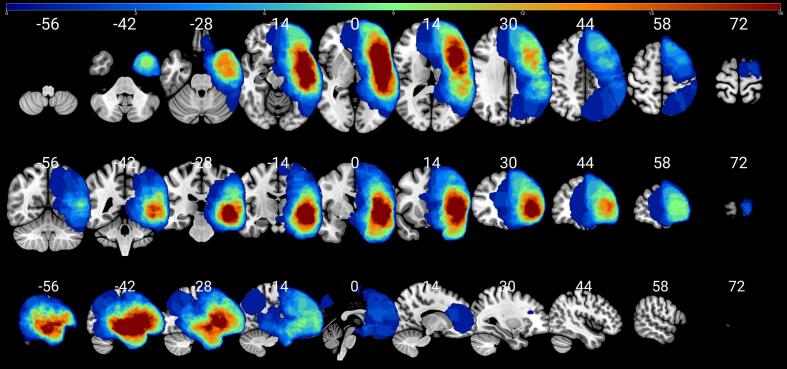
Lesion map. The figure shows the lesion map in MNI space with corresponding x (sagittal), y, (coronal) and z (axial) coordinates above each slice. All lesions are located in the perisylvian area. The color bar indicates the number of tumors per voxel.

In order to optimize the registration process to MNI ICBM 152 space, all anatomical T1 data sets were skull-stripped applying the ANTs brain extraction tool in combination with the public ANTs/ANTsR IXI brain template (https://doi.org/10.6084/m9.figshare.915436.v2) prior to MNI space registration (Avants et al., 2011). Furthermore, semi-automated lesion segmentations were generated with ITK-Snap and used as binarized masks (Yushkevich et al., 2006), see Fig. 1. The semi-automatic segmentation in ITK-SNAP relies on a two-step pipeline where first multiple image modalities used for segmentation are combined to produce a scalar image, followed by active contour segmentation and user-placed initiation seeds. The automated segmentation is divided into three steps. First, a probability map or a speed function is computed, based on the users preferences. Second, the user places one or more spherical seeds in the image for the segmentation, and third, the actual live contour evolution is initialized and the contour begins to evolve. Furthermore, the segmentations can be manually manipulated. (Yushkevich et al., 2016). All patients’ anatomical image data sets were registered to normalized space (MNI ICBM 152 non-linear 6th Generation Symmetric Average Brain Stereotaxic Registration Model) using the Advanced Normalization Tools (ANTs) software with the Symmetric Normalization (SyN) transformation model (Avants et al., 2011, Grabner et al., 2006). The registration matrix files were used to subsequently register the T1 data sets based rTMS coordinates to MNI ICBM 152 space as well. The rTMS coordinates were mapped to the Automated Anatomical Labeling (AAL3) parcellation (Rolls et al., 2020, Tzourio-Mazoyer et al., 2002) to define the grey matter regions of interest (ROI). The recently published AAL3 offers a whole brain parcellation of 170 ROIs (Rolls et al., 2020). The AAL3 coordinates were gathered using SPM12 and SPM-based viewing program xjView (Ashburner and Friston, 1999). After merging all rTMS spots, we calculated the error rate (ER) in each ROI. The ER was calculated by dividing the number of rTMS stimulations with error responses by the number of total rTMS stimulations within each ROI. To obtain detailed tumor locations, binary tumor masks were overlaid on AAL3 for grey matter and IIT human brain for white matter atlases. The IIT human brain atlas provides 42 probabilistic white matter tract masks (Zhang and Arfanakis, 2018). The percentages of overlap between tumor masks and atlas regions were calculated (Supplementary Table 1).

We calculated the ERs for each patient and subsequently mapped the ERs to AAL3 ROIs. Additionally, we calculated the number of stimulations per AAL3 ROI/patient. To increase the reliability of the ER results, the ERs derived from AAL3 ROIs which were stimulated less than 25% per AAL3 area per patient have been excluded. Given our clinical experience and the overall low incidence of rTMS induced language errors, we have defined a threshold of >=25% rTMS stimulations per AAL3 area as the minimum to draw meaningful conclusions about the functional relevance of a specific area in clinical decision making.

### 2.4 SVM

With the progression of computational power over the last two decades, SVM has received growing attention and is increasingly being used in biomedicine. SVMs are supervised machine learning models and aim to classify data points by maximizing the margin between classes (Cortes and Vapnik, 1995). SVM can be used for nonlinear classification using kernel tricks by implicitly mapping inputs to high-dimensional feature spaces using various kernel functions (Liu et al., 2019).

**Fig. 2:**
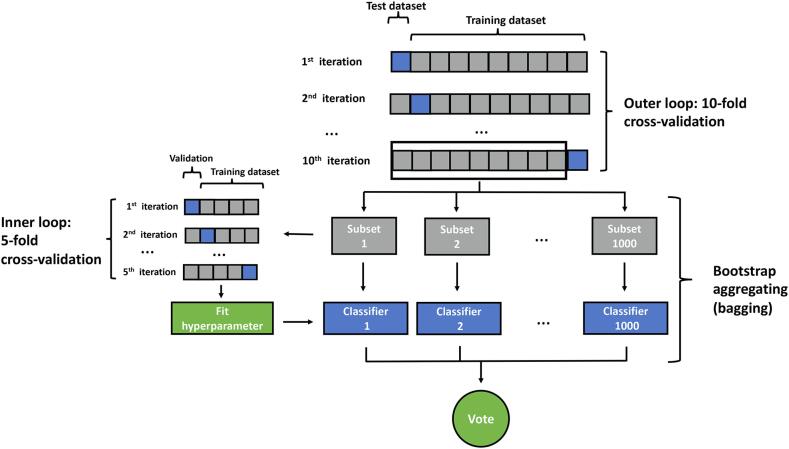
SVM analysis pipeline. Nested cross-validation and bootstrap aggregating (bagging).

In the present study we applied the linear kernel function using our data set to classify the patients into aphasic or non-aphasic groups. Missing data were interpolated with median imputation because the features were not normally distributed (SVM1). To ensure that missing data did not affect the SVM results, we applied a k-nearest neighbors algorithm (k-NN) imputation method in a second SVM model (SVM2) instead of a median imputation (Stewart et al., 2018). The preference for k-NN was based on the fact that with increasing number of missing values, the k-NN imputation becomes more robust to bias (Wu et al., 2019). In a third SVM model (SVM3), we excluded 26 patients because they did not receive rTMS over their right hemisphere. All patients’ TMS ERs in each AAL3 area, age, gender, tumor WHO grade and principal component analysis (PCA) components of individual IIT and AAL3 lesion percentages were fed into the SVM model as features (cf. 2.5 for PCA description). To effectively determine the meaningful features, recursive feature elimination was used for feature selection. Recursive feature elimination ranked the features, then recursively removed the less important features and built a model on those remaining features. A nested cross-validation (10-fold outer loop and 5-fold inner loop) approach was applied for training (Varoquaux et al., 2017). The nested cross-validation uses an internal cross-validation loop to adjust the parameters and select the best model. The outer cross-validation loop is used to evaluate models selected by the inner loop. The penalty parameter C was optimized by an internal cross-validation loop. In the inner loop the parameter C was tested from 2^−10^ to 2^10^ with a 0.1 step. Next, the data were proportionally split into 5 subsets. One was assigned to test the set and others were assigned to train the set. Subsequently, the optimized parameter C was estimated from the highest average classification accuracy. In our data, the ratio of aphasia to non-aphasia was 1 to 2, the proportion of patients in each fold was also 1 to 2 for both the inner and outer loop. Due to the unbalanced data, we adjusted the weight of the aphasia group to 2.0, the parameter C of the class aphasia was set to weight × C (Huang et al., 2013). To reduce the variance, we combined a model aggregation method called bagging and cross-validation. In each convolution, the data was resampled by random resampling (still according to the ratio of aphasia to non-aphasia of 1 to 2) so that 1000 training sets and corresponding models were generated and finally averaged (Poldrack et al., 2019). For machine learning coding, we used MATLAB R2014b (MathWorks, Natick, MA, US) with LIBSVM (Chang and Lin, 2011). We evaluated the performance of the SVM model by sensitivity, specificity, its overall accuracy, and the area under the receiver operating curve (AUC). The overall accuracy is the ratio of the correctly predicted classification of the entire cohort into the aphasic or non-aphasic group. The code and data used for SVM classification is archived as a MATLAB script on Zenodo (https://doi.org/10.5281/zenodo.3727663) and openly accessible (Wang et al., 2020). Fig. 2 demonstrates the SVM analysis pipeline.

### 2.5 Comparison of SVM and logistic regression results

**Supplementary figure 1:**
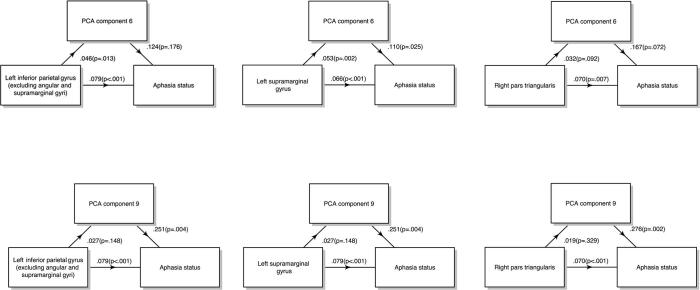


In order to detect the capability of tumor location to predict aphasia status, logistic regression was used to detect the relationship between region specific lesion percentages and aphasia status in a control analysis. As a first step of this analysis, the variance inflation coefficients of obtained lesion percentages were tested, because neighboring ROIs are likely to show correlations between their tumor overlaps and the resulting multicollinearity of tumor location variables would be problematic for logistic regression analysis (Supplementary Table 5). If the variance inflation coefficients were greater than 5, we used PCA to address the problem of multicollinearity (cf. 2.4). Only ROIs with sufficient lesion affection were included. Sufficient lesion affection was defined in the context of the current study as grey/white matter regions where the tumor has affected more than 10% of all voxels comprising that particular region in at least 5% of the patient sample (Sperber and Karnath, 2017). Resulting PCA component scores were subsequently entered as predictors in binary logistic regression analysis and fed into the SVM models, which also reduced the feature space for analysis (compared to entering individual AAL3 and IIT regions). To test whether the results obtained from the SVM model were influenced by tumor location, we subsequently performed a mediation analysis based on linear and binary logistic regression analyses (Supplementary Fig. 1).

### 2.6 Statistical analysis

Statistical analysis was performed by using MATLAB R2014b (MathWorks, Natick, MA, US) and SPSS22 (IBM SPSS, Armonk, New York, US). To compare continuous variables, two tailed Student’s *t*-tests or Mann–Whitney U tests were performed separately, depending on the normality of the data. Significant effects were considered at *p* < .05. Fisher’s exact test (for expected values less than five) or Pearson’s chi-squared test (larger values) were applied for the comparison of parameter variables. A Spearman's rank-order correlation was used to test the association between BAS and AAT T-scores. With respect to multiple comparison analyses, statistical significance values for ROIs of ER comparisons were adjusted by the Holm-Bonferroni method (Table 4).

### 2.7 Data availability

Parts of the data that support the findings of this study are not publicly available due to information that could compromise the privacy of the participants but are available from the corresponding author on reasonable request. However, the code we used is openly available under the following address (https://doi.org/10.5281/zenodo.3727663) and is cited at the corresponding passage (Wang et al., 2020).

## 3 Results

### 3.1 Patients

There was a strong, negative correlation between BAS and AAT T-scores, which was statistically significant (rs(60) = −0.732, p = 3.6593E−8). Twenty-nine (32.2%) of the recruited 90 patients presented with presurgical aphasic language disorders. The demographic data and comparison between aphasic and non-aphasic patients are provided in Table 1. There were no significant differences in gender or tumor size in relation to aphasia (χ^2^ = 1.207[1, 90], *p* = .272; *t*[88] = 0.023, *p* = .982 respectively). Aphasic patients were older (56.69 ± 13.64) than non-aphasic patients (45.13 ± 12.84), with a highly significant difference of *p* = .0003 (t [52] = 3.83). The tumor locations are shown in Supplementary Table 1.

### 3.2 Presurgical rTMS mapping

**Fig. 3:**
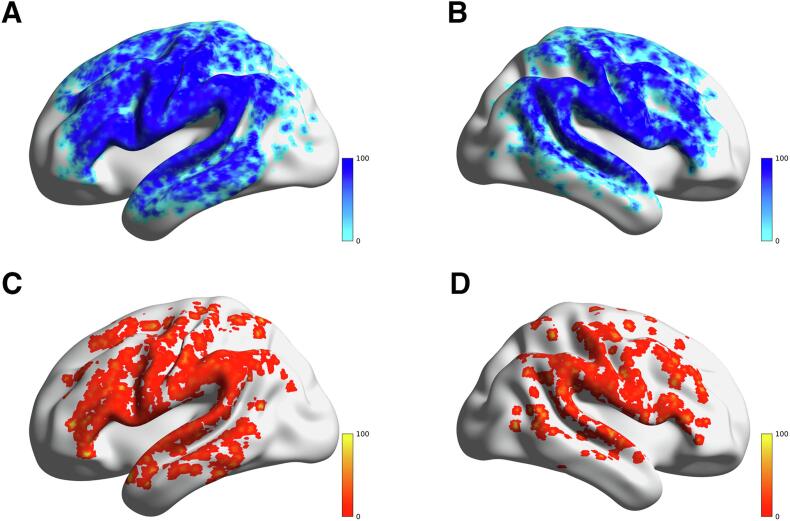
Percentages of voxel-wise rTMS stimulations of all patients in MNI space: (A) Left negative spots. (B) Right negative spots. (C) Left positive spots. (D) Right positive spots. The numbers and the color bar indicate the percentages of rTMS positive or negative stimulations per voxel.

Presurgical rTMS speech mapping was successful in all patients and generally well tolerated. Twenty-six (19 non-aphasic, 7 aphasic) patients were only mapped on their left hemisphere due to fatigue or decreasing level of attention. The mapping results of frontopolar and temporopolar cortices were not considered for analysis due to the discomfort evoked by the rTMS mapping in these areas (Fig. 3). The number of missing ER data points across all patients’ AAL3 ROIs was 28.8% while 82.2% of patients showed missing ER data points.

The mean VAS score during rTMS mapping was 3.9 ± 2.9 in the left and 3.7 ± 2.8 in the right hemisphere. The ER of the entire brain mapping in the aphasia group (Mdn = 7.49) was significantly higher (p < .0001, Z = 4.60, η^2^ = 0.24) than the ER of the entire brain mapping in the non-aphasia group (Mdn = 3.48).

Further, the aphasic patients showed a significantly higher ER in their left hemisphere compared to the non-aphasic patients and a non-significant difference of ER in their right hemisphere (Table 1). The rTMS spots were categorized into positive and negative spots. rTMS positive spots indicate that the rTMS stimulation caused an error response of any type, whereas a negative spot would indicate no error response. The overall rTMS positive spot distribution showed no clear pattern, not favoring specific cortical areas (Fig. 3). Moreover, the closely matching overall cortical distribution of both positive and negative rTMS spot distribution further demonstrates the non-occurrence of a particular pattern. We provide detailed results regarding the individual error rates of rTMS, individual numbers of positive rTMS stimulations and individual numbers of the sum of rTMS stimulations per AAL3 area in Supplementary Tables 2-4.

### 3.3 AAL3 and IIT labelling

**Fig. 4:**
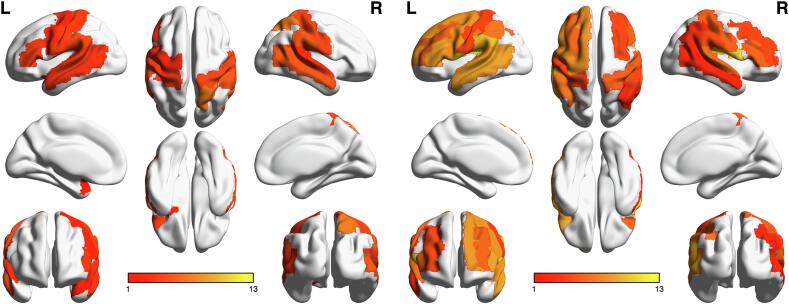
Visualization of overall ER distribution in relation to AAL3 parcellation in non-aphasic (left) and aphasic (right) groups. The color bars indicate the median ER per area.

The analysis of aphasic and non-aphasic patients in relation to rTMS ERs distributions showed specific cortical patterns. The numbers of stimulations per AAL3 ROI/patient were M = 14.9, SD = 16.2. ERs derived from AAL3 ROIs which were stimulated less than 6 times (< = 25% of stimulations per AAL3 ROI/patient) have been excluded. The threshold of > = 25% resulted in a total inclusion of 28 AAL3 ROIs per patient (Fig. 4 & Table 2).

**Table 2:** ER distribution in non-aphasic and aphasic patients.

Region	Overall ER (%) Mdn (IQR)		Missing subjects n (%)	
	Non-aphasic patients	Aphasic patients	Non-aphasic patients	Aphasic patients
Left angular gyrus	0 (0)	0 (11.11)	19 (31.15)	9 (31.03)
Right angular gyrus	0 (0)	2.63 (12.5)	28 (45.90)	13 (44.83)
Left inferior frontal gyrus, opercular part	0 (8.33)	7.69 (14.29)	7 (11.48)	4 (13.79)
Right inferior frontal gyrus, opercular part	0 (9.76)	0 (11.46)	22 (36.07)	9 (31.03)
Left pars triangularis	3.70 (7.42)	8.71 (8.26)	6 (9.84)	5 (17.24)
Right pars triangularis	0 (6.63)	5.88 (14.28)	22 (36.07)	8 (27.59)
Left middle frontal gyrus	0 (7.41)	5.88 (10.71)	12 (29.67)	8 (27.59)
Right middle frontal gyrus	0 (8.90)	4.45 (10.63)	23 (37.70)	9 (31.03)
Left superior frontal gyrus, dorsolateral	0 (2.80)	8.33 (20)	29 (47.54)	17 (58.62)
Right superior frontal gyrus, dorsolateral	0 (0)	0 (10)	34 (55.74)	16 (55.17)
Left inferior parietal gyrus (excluding angular and supramarginal gyri)	0 (6.25)	7.68 (9.88)	8 (13.11)	3 (10.34)
Right inferior parietal gyrus (excluding angular and supramarginal gyri)	0 (0)	2.94 (14.88)	21 (24.43)	9 (31.03)
Left superior parietal gyrus	0 (11.65)	0 (0)	27 (44.26)	13 (44.83)
Right superior parietal gyrus	6.97 (9.09)	0 (5)	31 (50.82)	14 (48.28)
Left postcentral gyrus	3.85 (6.73)	4.08 (11.24)	4 (6.58)	2 (6.90)
Right postcentral gyrus	4.35 (7.69)	5.56 (9.98)	20 (32.79)	7 (24.14)
Left precentral gyrus	2.50 (6.61)	8.02 (11.60)	7 (11.48)	3 (10.34)
Right precentral gyrus	0 (5.56)	5 (14.29)	20 (32.79)	8 (27.59)
Left rolandic operculum	0 (2.85)	13.39 (17.5)	7 (11.48)	5 (17.24)
Right rolandic operculum	0 (0)	11.11 (15.38)	20 (32.79)	8 (27.59)
Left supramarginal gyrus	4.55 (8.33)	10 (7.69)	8 (13.11)	4 (13.79)
Right supramarginal gyrus	5.26 (10.39)	4.55 (10)	19 (31.48)	8 (27.59)
Left middle temporal gyrus	3.46 (8.35)	8.70 (7.5)	9 (14.75)	0 (0)
Right middle temporal gyrus	5.13 (8.52)	2.63 (11.92)	25 (40.98)	15 (51.72)
Left temporal pole: superior temporal gyrus	0 (8.68)	0 (0)	16 (26.23)	10 (34.38)
Right temporal pole: superior temporal gyrus	0 (0)	0 (6.25)	30 (49.18)	10 (34.48)
Left superior temporal gyrus	3.18 (9.09)	7.94 (9.24)	3 (4.92)	1 (3.45)
Right superior temporal gyrus	3.13 (7.14)	6.25 (8.17)	20 (32.79)	7 (24.14)

Note: Only AAL3 ROIs that were stimulated at least 6 times were considered for the calculation of ERs.

### 3.4 SVM classification

**Fig. 5:**
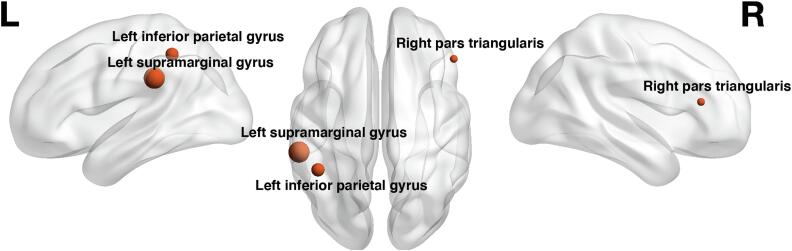
Visualization of spherical ROIs-based SVM (SVM1) weights. The visualization illustrates the different weights of ROIs classified by the SVM (SVM1) model, shown in left sagittal, dorsal and right sagittal views. The ROI sizes indicate the SVM (SVM1) weights of the AAL3 areas.

**Fig. 6:**
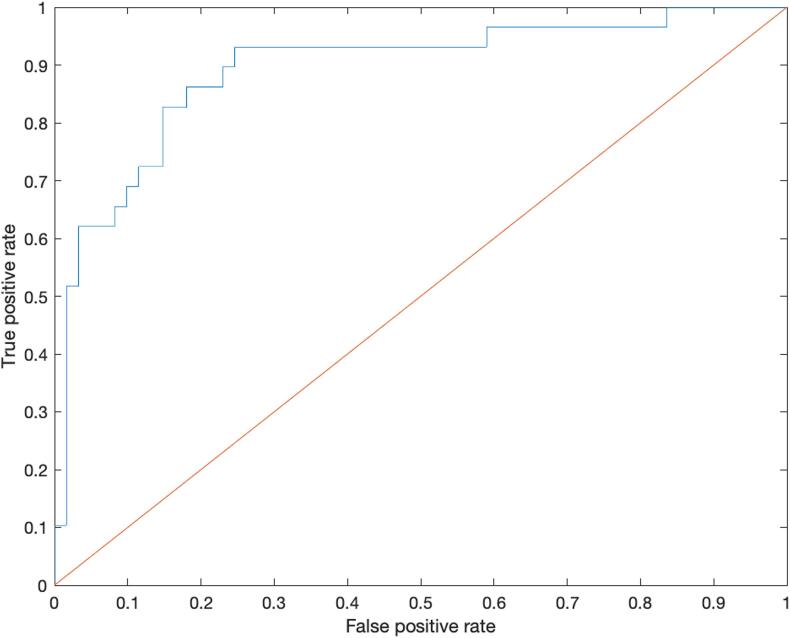
ROC for the SVM (SVM1) model with AUC = 85.4%. The ROC curve shows the true positive rate against the false positive rate at various threshold settings.

**Fig. 7:**
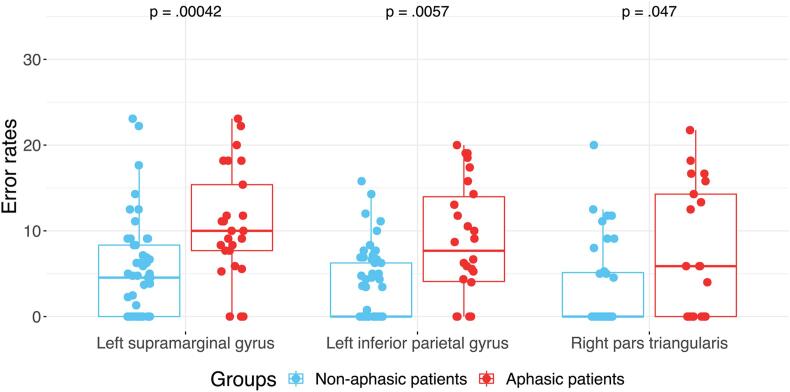
ER comparison between aphasic and non-aphasic groups in relation to SVM (SVM1)-derived AAL3 ROIs.

**Supplementary figure 2:**
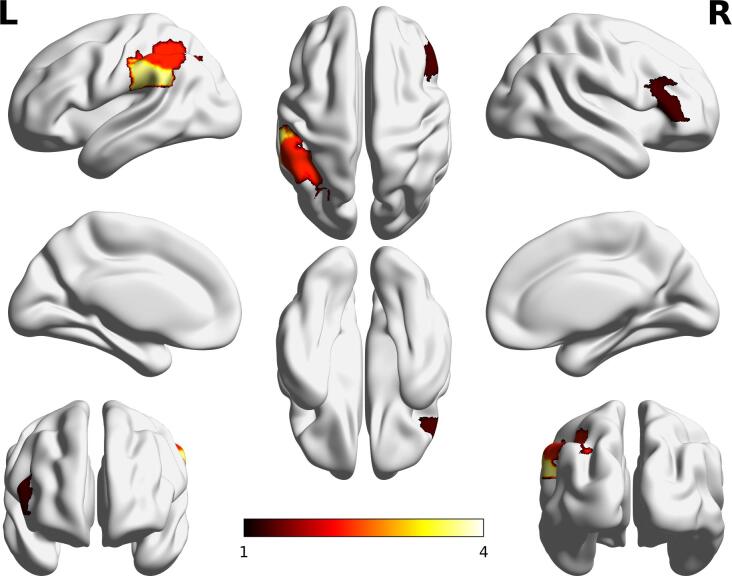

All 28 ER ROIs, age, gender, tumor WHO degree and tumor location PCA components were taken as input features for the SVM-recursive feature elimination (RFE) model to classify the aphasia status. The best classifier of final accuracy of the classification for aphasic and non-aphasic tumor patients were 85.53% (SVM1), 82.4% (SVM2) and 77.6% (SVM3). After RFE was embedded within a 10-fold cross-validation framework, four features were selected as the most important based on the weight. These four features were age, ER of right pars triangularis, ER of left supramarginal gyrus and ER of left inferior parietal gyrus (excluding angular and supramarginal gyri). All models (SVM1-SVM3) yielded the same four features after RFE. Their weights are illustrated in Fig. 5 (and Supplementary Fig. 2) and Table 3. The sensitivities of the models were 86.2% (SVM1), 90.0% (SVM2) and 59.1% (SVM3), the specificities were 82.0% (SVM1), 78.7% (SVM2) and 85.7% (SVM3) and the AUC’s were 89.3% (SVM1), 86.7% (SVM2) and 74.8% (SVM3). Fig. 6 illustrates the model’s receiver operating characteristic (ROC) curve and shows the AUC. The ERs of these three ROIs showed significant differences between the aphasic and non-aphasic groups after false discovery rate (FDR) correction (see Table 4 and Fig. 7). A mediation analysis revealed that the tumor location did not explain the full predictive value of TMS induced ER for aphasic status (Supplementary Fig. 1).

**Table 3:** Weights learned from SVM classification with LibSVM, linear kernel.

Feature	Weight
Left inferior parietal gyrus (excluding angular and supramarginal gyri)	2.28
Left supramarginal gyrus	3.64
Right pars triangularis	1.34
Age	2.98

**Table 4:** Error rate comparisons between aphasic and non-aphasic patients in the AAL3 areas with SVM weights.

Area	Overall ER (%) Mdn (IQR)		*p*	*Z-score*	*η^2^*	Number of patients	
	Non-aphasic patients	Aphasic patients				Non-aphasic patients	Aphasic patients
Left inferior parietal gyrus (excluding angular and supramarginal gyri)	0 (6.25)	7.68 (9.88)	0.0057	3.448	0.152	53	26
Left supramarginal gyrus	4.55 (8.33)	10 (7.69)	0.00042	3.794	0.182	53	25
Right pars triangularis	0 (5.00)	5.88 (14.28)	0.047	1.984	0.069	39	21

Note: Note: Only AAL3 ROIs that were stimulated at least 6 times were considered for the calculation of ERs.

Number of tests = 3. Results computed by Matlab multicmp and ranksum toolboxes.

*η^2^* represents the effect size of Mann-Whitney *U* test: *η^2^* = Z^2^/N.

### 3.5 Logistic regression and mediation analyses

To calculate the overlap between tumors and ROIs, we included 194 ROIs that resulted from the AAL3 (170) and IIT (24) parcellations. Of the total 194 ROIs, 69 (AAL3, 48 ROIs; IIT, 21 ROIs) overlapped with tumors. The variance inflation coefficients of all overlap ratios were higher than 5 in all 69 ROIs, revealing strong multicollinearity between overlap ratios. After performing PCA, 10 PCA components were extracted (Kaiser-Meyer-Olkin value = 0.717; 10 components PCA revealed with an eigenvalue greater than 1; cumulative percentage variance = 89.2%; varimax rotation was used to achieve orthogonality of components, see rotated components matrix in Supplementary Table 5). A Kaiser-Meyer-Olkin value greater than 0.5 revealed eligibility for PCA. To explore the effect of tumor location on aphasia status, a logistic regression on these 10 PCA components to classify the aphasia status showed that only component 6 (including left Heschl’s gyri, left middle longitudinal fasciculus, left Rolandic operculum and left superior temporal gyrus) and PCA component 9 (including left middle temporal gyrus and left vertical occipital fasciculus) led to significant results (p = .023, Exp(B) = 1.85; p = .005, Expo(B) = 2.36; Table 5).

**Table 5:** Logistic regression on aphasia status and PCA components derived from overlapped ratio of ROIs (AAL3 and IIT).

	B	S.E.	Wald	df	Sig.	Exp(B)	95% C.I.for EXP(B)	
							Lower	Upper
Component1	−0.074	0.276	0.072	1	0.788	0.928	0.540	1.595
Component2	−0.036	0.256	0.019	1	0.889	0.965	0.584	1.595
Component3	0.115	0.242	0.227	1	0.634	1.122	0.698	1.805
Component4	0.187	0.258	0.524	1	0.469	1.206	0.727	2.001
Component5	0.511	0.271	3.549	1	0.060	1.666	0.980	2.835
Component6	0.617	0.272	5.144	1	0.023	1.854	1.087	3.161
Component7	0.006	0.247	0.001	1	0.979	1.006	0.620	1.633
Component8	−0.093	0.271	0.118	1	0.731	0.911	0.536	1.549
Component9	0.859	0.304	7.991	1	0.005	2.361	1.301	4.282
Component10	−0.359	0.311	1.333	1	0.248	0.698	0.380	1.285
Constant	−0.853	0.264	10.403	1	0.001	0.426		

## 4 Discussion

In the present study, we examined rTMS based language mapping by using an object naming task and group analysis in a spatially normalized cohort of 90 glioma patients. After spatial normalization, a linear SVM model using TMS ERs, tumor location and demographic data was applied to classify the patients' aphasia status. Regarding the SVM results, the findings of the present study can be linked to current neuroplasticity models from research in post-stroke aphasia, for example the use of spare capacity within or between networks via variable neuro-displacement (variable neuro-displacement is described as the process by which a neural network uses its free capacity and increases its activity and/or performance in more demanding conditions – it aims at titrating performance against energy costs) (Hartwigsen and Saur, 2019, Stefaniak et al., 2019). Moreover, similar to other tumor-induced language neuroplasticity studies, our results reveal an involvement of right pars triangularis in relation to aphasia caused by left-hemispheric lesions (Piai, 2019, Thiel et al., 2005). Regarding the SVM’s model underlying TMS spot distribution, the pattern of overall positive rTMS spots does not differ from the pattern of overall negative rTMS spots. Furthermore, the rTMS-based analysis demonstrated a bihemispheric perisylvian distribution of rTMS-positive cortical areas indicating a bilateral representation of language function. This bilateral susceptibility to rTMS disruption of language processing is less pronounced in rTMS studies on healthy volunteers, indicating functional reorganization of the language network in brain tumor patients (Rosler et al., 2014). This observation is in line with other studies, suggesting a pivotal role of left and right frontal, as well as left precentral, central and parietal areas for language function (Stefaniak et al., 2019). The wide-spread distribution of rTMS positive responses is consistent with the large-scale distributed network configuration of language.

### 4.1 SVM classification

The SVM model results demonstrate the accuracy of patient classification into different groups (i.e. aphasic, non-aphasic) based on distributions of rTMS language mapping error rates. The SVM results show that right pars triangularis, left supramarginal gyrus, left inferior parietal gyrus and age contributed more to the classification model than rTMS language error rates in other areas and patients’ lesion profile features. The important contribution of rTMS-induced object naming error rates in right pars triangularis to distinguish aphasic from non-aphasic tumor patients could plausibly be the result of a functional shift of language abilities from the left to the right hemisphere to compensate for the initial impact of left-hemisphere brain tumors on the language network. However, this may be caused by behavioral variability due to differences in premorbid functions of certain brain areas, or because of differences in the brain’s potential for reorganization to compensate for lost function (Price, 2018).

The overall increase of errors in aphasic patients shows that even after baseline correction of the object-naming image stack, patients with aphasia make more errors during mapping in the left hemisphere. We used a cut-off value of 28% error rate during baseline naming of the object naming task (Schwarzer et al., 2018). Yet, concerning the aphasic patients of the present study, the SVM results demonstrate that the overall increase of ERs over both hemispheres manifests in a disproportional increase of errors in the right pars triangularis. This finding plausibly points towards an increased involvement in object naming of the right hemisphere due to the left hemisphere pathology. Further, it points towards the previously claimed object naming susceptibility of the right hemisphere in areas such as the right inferior frontal gyrus (Neininger and Pulvermuller, 2003, Rosler et al., 2014, Thiel et al., 2005). In stroke studies recruitment of the right inferior frontal gyrus after left hemispheric stroke was associated with both, favorable and unfavorable outcomes compared to cases with predominant regional ipsilateral recruitment (Hartwigsen and Saur, 2019). TMS-induced suppression of the right pars triangularis in patients with post-stroke aphasia has previously been associated with improvements in object naming performance (Naeser et al., 2011). This finding has been replicated a number of times (Barwood et al., 2013, Harvey et al., 2019, Kindler et al., 2012, Thiel et al., 2013), although other studies have reported substantial inter-patient variability in the effect of inhibitory TMS over the right pars triangularis in acute stroke patients (Seniow et al., 2013, Winhuisen et al., 2005). Yet, in brain tumor patients, there is evidence that a shift towards the right hemisphere is associated with better outcome after surgery in the left hemisphere, supporting hypotheses of increased functional reserve in patients with more bilateral distributions of language function (Ille et al., 2016). The current results are compatible with both, a beneficial or a deteriorating effect of right hemispheric neuroplasticity on aphasia status. The difference in right hemispheric error rates could demonstrate neuroplasticity compensating for (otherwise more severe) language impairments following tumor induced lesions. Alternatively, at least in theory, the right hemispheric neuroplasticity could also reflect a dysfunctional mechanism which leads to (an increase of) aphasics symptoms in the patients investigated in the current analysis and could thus explain the SVM classification findings.

Regarding the effect of tumor location, PCA component 6 (including left Heschl’s gyri, left middle longitudinal fasciculus, left Rolandic operculum and left superior temporal gyrus), PCA component 9 (including left middle temporal gyrus and left vertical occipital fasciculus) and the related mediation effect, and thus the lesion topographies, may explain some of the predictive values of the ERs for the aphasia status, but critically not in its entirety. Therefore, the ERs in right pars triangularis, left supramarginal gyrus and left inferior parietal gyrus can be seen to be informative for the aphasia status, independent of the influence of the individual lesion topography. In contrast, the feature of age contributed significantly to the SVM model. This result is confirmed by an earlier study predicting language dysfunction, that reported correlation of age and tumor grade with aphasia, but no correlation of tumor location (Recht et al., 1989). Even years later it is argued that the location of the tumor does not correlate with the type of aphasia or its severity before, during and after tumor resection (Davie et al., 2009). This supports the notion that general tumor induced network disconnection is relevant to aphasia and not necessarily related to specific lesion locations. Regarding the feature of age, our results may support the decreasing potential for neuroplasticity with age, as has been shown in previous studies (Lu et al., 2004). However, this was not directly tested in this study.

Importantly, our results confirm the importance of the left supramarginal gyrus and left inferior parietal gyrus for maintaining speech function, as it has already been shown in early lesion mapping studies (Penfield and Roberts, 1959). Additionally, lesions disconnecting traditional Broca's and Wernicke's areas, including the left supramarginal gyrus and left inferior parietal gyrus, cause different syndromes of clinical aphasias (Catani et al., 2005). Our findings regarding the importance of the left supramarginal gyrus could be linked to Geschwind's territory that connects traditional Broca's and Wernicke's areas via the arcuate fascicle with its anterior and posterior segments (Catani et al., 2005). The supramarginal and angular gyri represent high risk areas, an indication of low functional resectability, which further highlights their crucial role in relation to aphasia (Ius et al., 2011).

## 5 Limitations

The number of rTMS stimulations per area is heterogenous, with the discomfort evoked by the current rTMS methodology limiting cortical coverage. By and large, only one speech task was used in the study, namely object naming. This may lead to a systematic error, since different cortex areas may have different sensitivities to certain linguistic submodalities (De Witte et al., 2015), but also due to a possible location specificity (Krieg et al., 2017). Further, an object naming task alone provides only a partial picture of language processing. In addition, aphasic patients with an error rate of more than 28% in the baseline object naming task were excluded from rTMS mapping, biasing the sample to patients with milder forms of aphasia. Also, error annotation is user dependent, leading to difficulties when comparing results across institutions and establishing objective measures for the analysis of language performance. Moreover, the impact of aphasia severity was not tested and might be addressed in another study with larger subgroup analyses. It should also be mentioned that cognitive mapping depends on the patient’s individual performance. This leads to a high degree of variability in cognitive mapping, which is difficult to control for. Finally, we would like to state that the results are atlas dependent and may therefore be compromised by the choice of parcellation scheme.

## 6 Conclusion

The results of the present study based on group analysis, cortical parceling and machine learning classification with an SVM model in 90 patients suffering from left perisylvian glioma, show that the pattern of rTMS-induced errors in aphasic patients differs distinctly from the pattern in non-aphasic patients. Further, our findings demonstrate that patients with aphasia due to left perisylvian brain tumours have a generally increased area of right perisylvian rTMS error susceptibility, particularly in the right pars triangularis, as well as a larger right perisylvian distribution of ERs. This finding points towards a stronger, possibly essential, involvement of the right frontal lobe as a result of aphasia-induced functional reorganization. While reliable non-invasive mapping of the functional language-network remains a major challenge in individual brain tumor patients, the results of this study could suggest that machine learning adds to the detection of distinct patterns of functional reorganization in patients with language eloquent brain tumors. To the best of our knowledge, this study constitutes the first machine learning based classification of rTMS language mapping results of tumor patients.

## Declaration of Competing Interest

The authors declare that they have no known competing financial interests or personal relationships that could have appeared to influence the work reported in this paper.

