# Supplementary material for: Support vector machine based aphasia classification of transcranial magnetic stimulation language mapping in brain tumor patients
Source: Neuroimage Clin. 2020 Dec 24;29:102536. doi: 10.1016/j.nicl.2020.102536 (PMC7772815; doi:10.1016/j.nicl.2020.102536)
Supplement: Supplementary data 1 [file mmc1.docx]

Supplementary table 1. Coverage ratio between tumors and atlas-based ROIs in non-aphasic group and aphasic groups.

| Label | Non-aphasic group | | | |  | Aphasic group | | | |
| --- | --- | --- | --- | --- | --- | --- | --- | --- | --- |
|  | Mdn | IQR | Min | Max |  | Mdn | IQR | Min | Max |
| Precentral_L | $0$ | $0$ | $0$ | $76.2$ |  | $0$ | $0.7$ | $0$ | $33$ |
| Frontal_Sup_2_L | $0$ | $0.8$ | $0$ | $83.9$ |  | $0$ | $0$ | $0$ | $88.1$ |
| Frontal_Mid_2_L | $0$ | $9.4$ | $0$ | $81.4$ |  | $0$ | $0$ | $0$ | $97.9$ |
| Frontal_Inf_Oper_L | $0.1$ | $15.9$ | $0$ | $95.3$ |  | $0$ | $31.8$ | $0$ | $98$ |
| Frontal_Inf_Tri_L | $0$ | $36.3$ | $0$ | $92.7$ |  | $0$ | $7.7$ | $0$ | $98.8$ |
| Frontal_Inf_Orb_2_L | $0$ | $22.5$ | $0$ | $96.2$ |  | $0$ | $3.7$ | $0$ | $98$ |
| Rolandic_Oper_L | $0.2$ | $14.9$ | $0$ | $96.2$ |  | $2.9$ | $36.2$ | $0$ | $90.1$ |
| Olfactory_L | $0$ | $0$ | $0$ | $1$00 |  | $0$ | $0$ | $0$ | $43.8$ |
| Frontal_Sup_Medial_L | $0$ | $0$ | $0$ | $97.3$ |  | $0$ | $0$ | $0$ | $82.5$ |
| Frontal_Med_Orb_L | $0$ | $0$ | $0$ | $95.2$ |  | $0$ | $0$ | $0$ | $56.1$ |
| Rectus_L | $0$ | $0$ | $0$ | $94.9$ |  | $0$ | 0 | $0$ | $46.9$ |
| OFCmed_L | $0$ | $0$ | $0$ | $92.9$ |  | $0$ | $0$ | $0$ | $72.6$ |
| OFCant_L | 0 | $0$ | $0$ | $93.5$ |  | $0$ | $0$ | $0$ | $93.1$ |
| OFCpost_L | $0$ | $2.2$ | $0$ | $97.7$ |  | $0$ | $14.9$ | $0$ | $71.6$ |
| OFClat_L | $0$ | $0$ | $0$ | $95.2$ |  | $0$ | $0$ | $0$ | $99$ |
| Insula_L | $8.5$ | $31.5$ | $0$ | $98.6$ |  | $20.2$ | $32.3$ | $0$ | $90.2$ |
| Hippocampus_L | 0 | $0$ | $0$ | $88.9$ |  | $0.4$ | $20.9$ | $0$ | $90.1$ |
| ParaHippocampal_L | $0$ | $0$ | $0$ | $63.6$ |  | $0$ | $6.4$ | $0$ | $87.5$ |
| Amygdala_L | $0$ | $0$ | $0$ | $1$00 |  | $0$ | $0.8$ | $0$ | $100$ |
| Fusiform_L | $0$ | $0$ | $0$ | $55.7$ |  | $0$ | $11$ | $0$ | $44.5$ |
| Postcentral_L | $0$ | 3 | $0$ | $30$ |  | $0$ | $0.4$ | $0$ | $30.6$ |
| Parietal_Sup_L | $0$ | $0$ | $0$ | $71.9$ |  | $0$ | $0$ | $0$ | $0$ |
| Parietal_Inf_L | $0$ | $0$ | $0$ | $93.3$ |  | $0$ | $0$ | $0$ | $21.8$ |
| SupraMarginal_L | $0$ | $0.4$ | $0$ | $95.8$ |  | $0$ | $0$ | $0$ | $85.6$ |
| Angular_L | $0$ | $0$ | $0$ | $99.5$ |  | $0$ | $0$ | $0$ | $9.8$ |
| Caudate_L | $0$ | $0$ | $0$ | $85.3$ |  | $0$ | $1.6$ | $0$ | $25.6$ |
| Putamen_L | $0$ | $13.1$ | $0$ | $99.6$ |  | $2.3$ | $25.7$ | $0$ | $90.6$ |
| Pallidum_L | $0$ | $0$ | $0$ | $99.2$ |  | $0$ | $0.5$ | $0$ | $100$ |
| Heschl_L | $0$ | $3.5$ | $0$ | $95.8$ |  | $12.4$ | $40.9$ | $0$ | $99.6$ |
| Temporal_Sup_L | $3$ | $21.3$ | $0$ | $70.6$ |  | $13.7$ | $48.2$ | $0$ | $90.9$ |
| Temporal_Pole_Sup_L | $0$ | $8.5$ | $0$ | $94.6$ |  | $0$ | $8.6$ | $0$ | $94.3$ |
| Temporal_Mid_L | $0$ | $15.3$ | $0$ | $40$ |  | $13.4$ | $29$ | $0$ | $58.5$ |
| Temporal_Pole_Mid_L | $0$ | $0$ | $0$ | $97.4$ |  | $0$ | $0$ | $0$ | $95.8$ |
| Temporal_Inf_L | $0$ | $0.2$ | $0$ | $50.4$ |  | $2.8$ | $20.7$ | $0$ | $52.1$ |
| Thal_VA_L | $0$ | $0$ | $0$ | $56.4$ |  | $0$ | 0 | $0$ | $67.3$ |
| Thal_VL_L | $0$ | $0$ | $0$ | $66.6$ |  | $0$ | $0$ | $0$ | $49.5$ |
| Thal_VPL_L | $0$ | $0$ | $0$ | $88.2$ |  | $0$ | $0.6$ | $0$ | $64.6$ |
| Thal_LGN_L | $0$ | $0$ | $0$ | $1$00 |  | $0$ | $0$ | $0$ | $100$ |
| Thal_MGN_L | $0$ | $0$ | $0$ | $87.6$ |  | $0$ | $2.8$ | $0$ | $100$ |
| Thal_PuA_L | $0$ | $0$ | $0$ | $81.7$ |  | $0$ | $0$ | $0$ | $100$ |
| Thal_PuM_L | $0$ | $0$ | $0$ | $10.3$ |  | $0$ | 0 | $0$ | $67.6$ |
| Thal_PuI_L | $0$ | $0$ | $0$ | $41.7$ |  | $0$ | $0$ | $0$ | $98.1$ |
| ACC_sub_L | $0$ | $0$ | $0$ | $88.4$ |  | $0$ | $0$ | $0$ | $44.7$ |
| ACC_sub_R | $0$ | $0$ | $0$ | $86.9$ |  | $0$ | $0$ | $0$ | $0$ |
| ACC_pre_L | $0$ | $0$ | $0$ | $1$00 |  | $0$ | $0$ | $0$ | $62$ |
| ACC_pre_R | $0$ | $0$ | $0$ | $100$ |  | $0$ | $0$ | $0$ | $6.6$ |
| ACC_sup_L | $0$ | $0$ | $0$ | $91.2$ |  | $0$ | $0$ | $0$ | $92.7$ |
| ACC_sup_R | $0$ | $0$ | $0$ | $94.1$ |  | $0$ | $0$ | $0$ | $91.7$ |
| Vent_Str_L | $0$ | $0$ | $0$ | $100$ |  | $0$ | $0$ | $0$ | $53.8$ |
| SN_pr_L | $0$ | $0$ | $0$ | $48.7$ |  | $0$ | $0$ | $0$ | $51.5$ |
| AC | $0.2$ | $5.7$ | $0$ | $28.6$ |  | $6.7$ | $11$ | $0$ | $27.6$ |
| AF_L | $5.7$ | $11.1$ | $0$ | $37.4$ |  | $11.4$ | $11.3$ | $0$ | $45.9$ |
| AST_L | $2.9$ | $14$ | $0$ | $72.4$ |  | $1.2$ | $12.8$ | $0$ | $71.5$ |
| CCMid | $0.2$ | $3.5$ | $0$ | $22.5$ |  | $0.6$ | $1.4$ | $0$ | $21$ |
| CC_ForcepsMinor | $0$ | $4.2$ | $0$ | $73$ |  | $0$ | $0$ | $0$ | $50.5$ |
| CC | $2.2$ | $3$ | $0.1$ | $17$ |  | $4$ | $3.5$ | $0.1$ | $18$ |
| CST_L | $0.3$ | $2.4$ | $0$ | $18.8$ |  | $0.8$ | $6.5$ | $0$ | $30.7$ |
| C_L | $0.3$ | $4.9$ | $0$ | $49.7$ |  | $0.2$ | $4.4$ | $0$ | $53.9$ |
| FPT_L | $1$ | $6$ | $0$ | $23.9$ |  | $1.2$ | $8$ | $0$ | $30.5$ |
| F_L_R | $0$ | $0$ | $0$ | $12.8$ |  | $0$ | $0$ | $0$ | $10$ |
| IFOF_L | $4.8$ | $15.4$ | $0$ | $47$ |  | $17.3$ | $23$ | $0$ | $38.2$ |
| ILF_L | $1.8$ | $9.2$ | $0$ | $38.5$ |  | $14.8$ | $30.2$ | $0$ | $40.1$ |
| ML_L | $0$ | $0.7$ | $0$ | $31.9$ |  | $0.4$ | $2.4$ | $0$ | $26.7$ |
| MdLF_L | $0.8$ | $8$ | $0$ | $44.7$ |  | $14.8$ | $27.1$ | $0$ | $51.6$ |
| OPT_L | $0$ | $0.2$ | $0$ | $33.7$ |  | $1.1$ | $5.7$ | $0$ | $30.7$ |
| OR_L | $0.8$ | $6$ | $0$ | $39$ |  | $10.8$ | $24.6$ | $0$ | $39$ |
| PPT_L | $0.1$ | $1.4$ | $0$ | $17.8$ |  | $0.7$ | $5$ | $0$ | $26.9$ |
| SCP | $0.3$ | $0.8$ | $0$ | $11.3$ |  | $0.6$ | $1.4$ | $0$ | $8.7$ |
| SLF_L | $2.7$ | $9.8$ | $0$ | $33.2$ |  | $3$ | $8.9$ | $0$ | $37.1$ |
| STT_L | $0$ | $0.7$ | $0$ | $24$ |  | $0.6$ | $1.9$ | $0$ | $20.3$ |
| UF_L | $1.8$ | $20.2$ | $0$ | $71.4$ |  | $13.1$ | $20.4$ | $0$ | $53.7$ |
