# Supplementary material for: Support vector machine based aphasia classification of transcranial magnetic stimulation language mapping in brain tumor patients
Source: Neuroimage Clin. 2020 Dec 24;29:102536. doi: 10.1016/j.nicl.2020.102536 (PMC7772815; doi:10.1016/j.nicl.2020.102536)
Supplement: Supplementary data 6 [file mmc6.docx]

Supplementary table 4. Varimax rotated component matrix of the principal component analysis run on the overlapped ratio of ROIs (AALL3 and IIT).

|  | | | | | | | | | | |
| --- | --- | --- | --- | --- | --- | --- | --- | --- | --- | --- |
|  | Component | | | | | | | | | |
|  | 1 | 2 | 3 | 4 | 5 | 6 | 7 | 8 | 9 | 10 |
| Temporal_Pole_Mid_L | **.917** | -.025 | .023 | -.010 | -.050 | .128 | .136 | .039 | -.107 | .030 |
| Amygdala_L | **.895** | -.050 | .178 | -.034 | .059 | .051 | .082 | .257 | -.072 | .002 |
| Temporal_Pole_Sup_L | **.862** | -.073 | .158 | -.057 | -.095 | .222 | .170 | .100 | -.135 | -.033 |
| AC | **.824** | -.089 | .069 | -.028 | .278 | .288 | -.071 | .175 | .229 | -.093 |
| ParaHippocampal_L | **.806** | -.028 | -.043 | -.027 | .466 | -.016 | .024 | .101 | .025 | -.020 |
| UF_L | **.787** | .073 | .429 | -.085 | .094 | .217 | .044 | .233 | .046 | -.080 |
| Temporal_Inf_L | **.772** | -.086 | -.122 | -.071 | .095 | .044 | -.137 | -.060 | .465 | -.104 |
| Fusiform_L | **.750** | -.051 | -.122 | -.060 | .386 | -.089 | -.066 | -.037 | .311 | -.057 |
| Hippocampus_L | **.727** | -.040 | -.073 | -.004 | .599 | .108 | -.076 | -.045 | .080 | -.050 |
| Pallidum_L | **.615** | -.005 | .141 | .062 | .297 | .075 | .121 | .581 | -.089 | .007 |
| ILF_L | **.588** | -.125 | -.068 | -.010 | .282 | .543 | -.152 | -.049 | .453 | -.048 |
| C_L | -.011 | **.940** | .244 | .141 | .018 | -.101 | .054 | .058 | -.022 | -.040 |
| Frontal_Sup_2_L | -.070 | **.937** | .221 | .080 | -.053 | -.080 | -.004 | -.050 | -.063 | -.039 |
| Frontal_Sup_Medial_L | -.081 | **.935** | .104 | -.053 | -.023 | -.016 | -.062 | -.041 | -.071 | -.002 |
| Frontal_Mid_2_L | -.070 | **.897** | .261 | .134 | -.083 | -.117 | .161 | -.050 | -.037 | -.077 |
| CC_ForcepsMinor | -.052 | **.841** | .485 | -.065 | -.039 | -.054 | .098 | .032 | -.048 | -.006 |
| FPT_L | .073 | **.714** | .364 | .383 | .180 | -.064 | .270 | .154 | -.033 | -.027 |
| Caudate_L | -.040 | **.670** | -.070 | .038 | -.047 | -.039 | .139 | .585 | .026 | -.024 |
| AST_L | -.025 | **.665** | .038 | .506 | -.079 | -.105 | .412 | .055 | -.069 | -.171 |
| OFCant_L | .000 | .383 | **.885** | .001 | -.037 | -.043 | .039 | .003 | -.025 | .014 |
| OFClat_L | .010 | .321 | **.849** | -.035 | .010 | -.069 | .159 | -.088 | -.044 | -.006 |
| OFCmed_L | .130 | .099 | **.788** | -.009 | -.091 | -.017 | -.069 | .481 | -.029 | -.041 |
| Frontal_Inf_Orb_2_L | .117 | .288 | **.759** | -.144 | .008 | -.068 | .439 | .061 | -.094 | -.011 |
| Frontal_Med_Orb_L | -.040 | .402 | **.743** | -.004 | -.054 | -.024 | -.232 | .125 | -.057 | -.047 |
| OFCpost_L | .465 | .036 | **.640** | -.057 | -.013 | .024 | .229 | .426 | -.112 | -.008 |
| IFOF_L | .410 | .255 | **.575** | -.112 | .307 | .276 | .157 | .190 | .292 | -.079 |
| CST_L | .002 | .074 | -.023 | **.832** | .402 | .140 | -.028 | .048 | .055 | .230 |
| SLF_L | -.119 | .204 | -.023 | **.820** | .013 | .094 | .272 | .028 | -.001 | .389 |
| PPT_L | .044 | .113 | -.014 | **.773** | .478 | .105 | -.036 | .058 | .119 | .199 |
| Precentral_L | -.087 | .166 | -.112 | **.751** | -.142 | -.189 | .095 | -.042 | -.117 | -.344 |
| AF_L | -.036 | .301 | -.009 | **.700** | .051 | .244 | .350 | -.006 | .393 | .106 |
| Postcentral_L | -.119 | -.103 | -.086 | **.694** | -.065 | .209 | -.018 | -.062 | -.226 | .260 |
| Thal_LGN_L | .332 | -.042 | -.038 | .055 | **.860** | .123 | -.019 | -.026 | .121 | -.063 |
| Thal_VPL_L | .297 | -.037 | .013 | .184 | **.786** | .196 | .071 | .106 | -.036 | -.015 |
| OPT_L | .042 | -.082 | .017 | .339 | **.578** | .254 | -.107 | .023 | .268 | .496 |
| OR_L | .512 | -.107 | -.042 | .016 | **.542** | .465 | -.113 | -.032 | .384 | -.042 |
| Temporal_Sup_L | .246 | -.149 | -.065 | .096 | .067 | **.900** | -.037 | -.010 | .114 | .129 |
| Heschl_L | .201 | -.113 | .008 | .158 | .291 | **.799** | .220 | .086 | -.083 | .089 |
| MdLF_L | .180 | -.129 | -.033 | .152 | .372 | **.629** | -.156 | -.045 | .414 | .339 |
| Rolandic_Oper_L | .144 | -.095 | .038 | .370 | .115 | **.565** | .528 | .118 | -.229 | .002 |
| Frontal_Inf_Oper_L | .033 | .221 | .001 | .324 | -.101 | -.044 | **.795** | .068 | -.109 | -.182 |
| Frontal_Inf_Tri_L | -.038 | .530 | .341 | .006 | -.054 | -.129 | **.674** | -.058 | -.047 | -.059 |
| Insula_L | .387 | .065 | .248 | .035 | .073 | .244 | **.639** | .423 | -.183 | -.079 |
| Olfactory_L | .348 | -.046 | .349 | -.019 | -.072 | -.046 | -.041 | **.799** | -.046 | -.022 |
| Putamen_L | .493 | .086 | .186 | .053 | .251 | .116 | .381 | **.637** | -.113 | -.039 |
| VOF_L | .021 | -.129 | -.062 | -.014 | .133 | .027 | -.100 | -.074 | **.874** | .064 |
| Temporal_Mid_L | .307 | -.145 | -.117 | -.052 | -.009 | .563 | -.233 | -.069 | **.599** | -.013 |
| Parietal_Inf_L | -.089 | -.078 | -.029 | .201 | -.018 | -.031 | -.080 | -.025 | .050 | **.910** |
| SupraMarginal_L | -.172 | -.122 | -.077 | .268 | -.048 | .373 | -.129 | -.046 | -.066 | **.714** |

Variables with high loadings are in bold.
